# Macrobenthic molluscs from a marine - lagoonal environmental transition in Lesvos Island (Greece)

**DOI:** 10.3897/BDJ.4.e9541

**Published:** 2016-11-01

**Authors:** Athanasios Evagelopoulos, Drosos Koutsoubas, Vasilis Gerovasileiou, Nikolaos Katsiaras

**Affiliations:** ‡ Department of Marine Sciences, University of the Aegean, Mytilene, Greece; § Institute of Marine Biology, Biotechnology and Aquaculture, Hellenic Centre for Marine Research, Heraklion, Greece; | Institute of Oceanography, Hellenic Centre for Marine Research, Anavissos, Greece

**Keywords:** biodiversity, environmental stress gradient, confinement gradient, transitional waters, coastal lagoons, solar saltworks

## Abstract

**Background:**

This paper describes an occurence dataset, also including numerical abundance and biomass data, pertaining to the macrobenthic molluscan assemblages from a marine - lagoonal environmental transition. The study system was the soft-substrate benthoscape of the area of the Kalloni solar saltworks (Lesvos Island, Greece). Specifically, the study area extended from the infralittoral zone of the inner Kalloni Gulf (marine habitat) to the bottoms of the first two evaporation ponds of the Kalloni solar saltworks (lagoonal habitat). Bottom sediment samples (3 replicates) were collected with a Van Veen grab sampler (0.1 m^2^) at four sampling sites, along a 1.5 km long line transect that spanned the marine - lagoonal environmental transition. A total of four surveys were carried out seasonally in 2004.  A total of 39,345 molluscan individuals were sorted out of the sediment samples and were identified to 71 species, belonging to the Gastropoda (36), Bivalvia (34) and Scaphopoda (1) classes. Numerical abundance and wet biomass (with shells) data are included in the dataset.

**New information:**

The dataset described in the present paper partially fills a significant gap in the scientific literature: Because ecological research of coastal lagoons has seldom explicitly considered the marine - lagoonal habitats interface, there are no openly accessible datasets pertaining to the particular structural component of the transitional waters benthoscapes of the Mediterranean Sea. Such datasets could prove valuable in the research of the structure and functioning of transitional waters benthoscapes. The present dataset is available as a supplementary file (Suppl. material 1) and can also be accessed at http://ipt.medobis.eu/resource?r=kalloni_saltworks_phd.

## Introduction

Transitional waters (e.g. estuaries, coastal lagoons) may be considered as "ecotone ecosystems" that structurally and functionally link marine, continental and freshwater ecosystems along the coastline (Levin et al. 2001, Basset et al. 2012). Their existence along the interface between the sea and the land determines their abiotic environment, which is characterized by complex spatial gradients in structural features and pronounced temporal variability (Little 2000). The often extreme in magnitude and variability abiotic factors control the composition and spatial distribution of the biota (Barnes 1980, Guelorget and Perthuisot 1992, Barnes 1994).

Macrobenthic invertebrates are considered as a key group among the biota in all coastal aquatic ecosystems (Levin et al. 2001). Molluscs are an important component of the macrobenthic fauna of coastal lagoons (Barnes 1994). Their abundance and diversity are also important in the lower salinity ponds of solar saltworks (e.g. Britton and Johnson 1987, Evagelopoulos et al. 2008, Pavlova et al. 1998, Vieira and Amat 1996), where the habitat is considered to be essentially lagoonal. The response of molluscan communities to the environmental stress gradients has been extensively studied in coastal lagoons (e.g. Guelorget and Perthuisot 1992, Koutsoubas et al. 2000, Reizopoulou and Nicolaidou 2004, Rossi et al. 2006), as well as in the lower salinity ponds of solar saltworks (e.g. Evagelopoulos and Koutsoubas 2008, Evagelopoulos et al. 2008). Ecological research of coastal lagoons has seldom explicitly considered the marine - lagoonal habitats interface (but see de Wit 2011). However, the interfaces between habitat patches are considered to be among the primary structural and functional components of landscapes (Turner and Gardner 2015). Their importance lies in the fact that they may modulate flows of materials, energy, organisms or information across the landscape, potentially also affecting processes inside the interacting habitat patches (Pickett and Cadenasso 1995, Wiens et al. 1985).

This paper describes an occurence dataset, also including numerical abundance and biomass data, pertaining to the macrobenthic molluscan assemblages from the marine - lagoonal environmental transition at the area of Kalloni solar saltworks (Lesvos Island, Greece). The present dataset paper partially fills a significant gap in the scientific literature, as no openly accessible datasets from case studies of marine - lagoonal habitats interfaces have been published thus far and such datasets could prove valuable in the research of the structure and functioning of transitional waters benthoscapes.

## General description

### Purpose

This dataset was assembled in the framework of the PhD thesis of Dr. Athanasios Evagelopoulos (Department of Marine Sciences, University of the Aegean, Greece) (Evagelopoulos 2008).

## Project description

### Title

Macrobenthic molluscs from the marine-lagoonal environmental transition at the area of Kalloni saltworks (Lesvos Island, Greece).

### Personnel

Drosos Koutsoubas (PhD thesis supervisor, sample collection, taxonomic identification), Athanasios Evagelopoulos (sample collection, taxonomic identification, data management), Vasilis Gerovasileiou (sample collection, laboratory analysis), Nikolaos Katsiaras (sample collection, laboratory analysis), Andreas Alifragkis (sample collection, laboratory analysis), Ioannis Vasiliadis (sample collection, laboratory analysis), Asimenia Kostidou (sample collection).

### Study area description

A detailed description of the study area is provided by Evagelopoulos and Koutsoubas (2008) and can be summarized as follows: The study area is characterised by a shallow water column (approx. 0.5 m deep), a soft-substrate bottom and a benthic vegetation of macroalgae and phanerogams (e.g. *Cladophora* sp., *Enteromorpha* sp., *Gracilaria* sp., *Ruppia* sp.). The habitat types according to the environment ontology of EnvO (Buttigieg et al. 2013, Buttigieg et al. 2016) occuring in the study area included the "neritic sub-littoral zone" (sampling site 1), the "artificial channels" (site 2) and the "lagoons" (sites 3 & 4). Macroalgal mats were developed at the inlet and in the ponds during the late spring - early summer of 2004. Wind forcing together with the shallow depth of the water column were the main factors involved in the recurrent bottom sediment resuspension and the high water turbidity that wereoften observed in the study area. The bottom sediment at the inlet and the ponds was anoxic near its surface, due to the high productivity of the ecosystem. Macroalgal growth led to a dystrophic crisis incident in the study area in the summer of 2004. 

### Funding

This dataset was assembled in the framework of the PhD thesis of Dr. Athanasios Evagelopoulos, which was supported by a PhD scholarship from HERACLITUS: Research scholarships with priority in basic research (2nd Operational Programme for Education and Initial Vocational Training, 3rd CSF) that was funded by the Greek Ministry of Education and co-funded by the ESF (EU).

Authoring of the present data paper and data management and upload to the MedOBIS IPT were supported by the LifeWatchGreece infrastructure (MIS 384676), funded by the Greek  Government under the General Secretariat of Research and  Technology (GSRT), National Strategic Reference Framework (NSRF).

## Sampling methods

### Study extent

**Figure 1.**
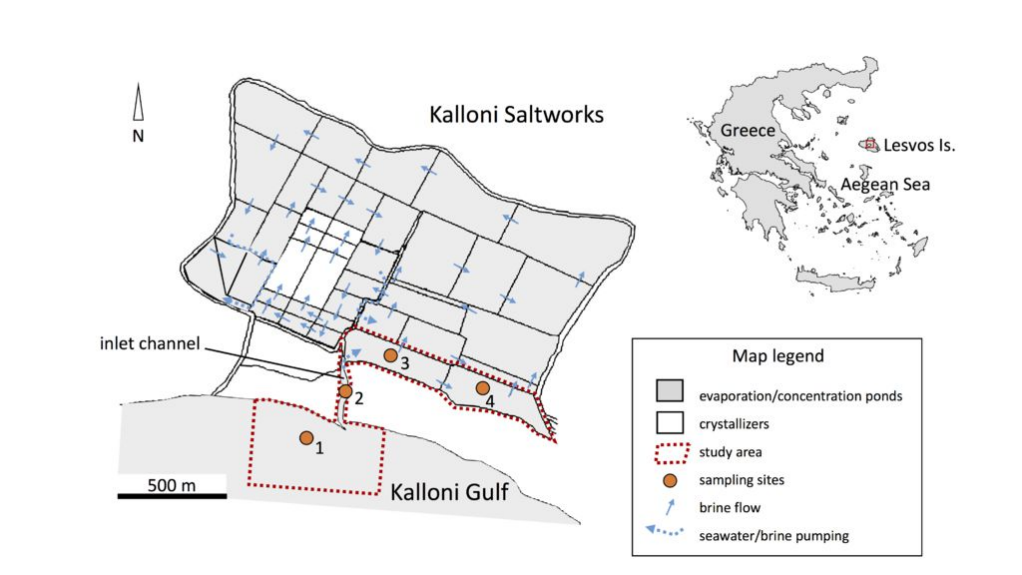
A map of the study area, indicating the sampling stations.

The study area (Fig. 1) consisted of the soft-substrate benthoscape of the area of the Kalloni solar saltworks (Lesvos Island, Greece). It extended from the infralitoral zone of the inner Kalloni Gulf (marine habitat) to the bottoms of the inlet and the first two evaporation ponds of the Kalloni solar saltworks (lagoonal habitat). The spatial extent of the study was thus approximatelly 1.5 km long, whereas its temporal extent was one year. The geographic coordinates of the sampling sites are given in Table 1.

### Sampling description

The field and laboratory methodology used followed the one described by Eleftheriou and Moore (2005). Bottom sediment samples (3 replicates) were collected with a Van Veen grab sediment sampler (0.1 m^2^ sampling surface area) at four sampling sites that were located along a line transect that spanned the marine-lagoonal environmental transition. The sampling sites (Fig. 1) were located at the infralittoral zone of the inner Kalloni Gulf (site 1), at the inlet channel of the saltworks (site 2) and at the first two evaporation ponds of the saltworks (sites 3 and 4, respectively). The surveys were seasonal and carried out in February, May, September and November of 2004.

### Quality control

- Species identification was reviewed by a molluscan taxonomy expert (Prof. Drosos Koutsoubas) and the species names were checked using the Taxon Match tool of the World Register of Marine Species  (http://www.marinespecies.org/aphia.php?p=match). Dimitra Mavraki and Matina Nikolopoulou of the LifeWatchGreece Core Team assisted in the management of data and the upload of the dataset to the MedOBIS IPT according to the DarwinCore schema (DwC).

### Step description

After their collection, the sediment samples were washed on-site through a 0.5 mm mesh sieve and subsequently fixed with 5% formalin and stained with Rose Bengal. In the laboratory, the macrobenthic invertebrates were sorted out of the sediment, and classified first into families and then into species. The individuals of each species in each sample were enumerated and their total wet weight (with shells) was measured with a high precision (0.1 mg) balance. During the data management process, the species names were checked with WoRMS, the dataset was prepared according to the Darwin Core standard and, finally, the data were uploaded to the MedOBIS IPT.

## Geographic coverage

### Description

The geographic coverage of the dataset extends from the part of the inner Kalloni Gulf that is adjacent to the Kalloni solar saltworks to the first two evaporation ponds of the saltworks. A map of the study area is given in Fig. 1 and the geographic coordinates of the sampling sites are given in Table 1.

### Coordinates

39.2 and 39.22 Latitude; 26.23 and 26.27 Longitude.

## Taxonomic coverage

### Description

The taxonomic coverage of the dataset is limited to the molluscan assemblages of the study area. Specifically, the dataset includes gastropod, bivalve and scaphopod species. A list of the species included in the dataset, also indicating their taxonomic classification, is given in Table 2.

**Figure 2.**
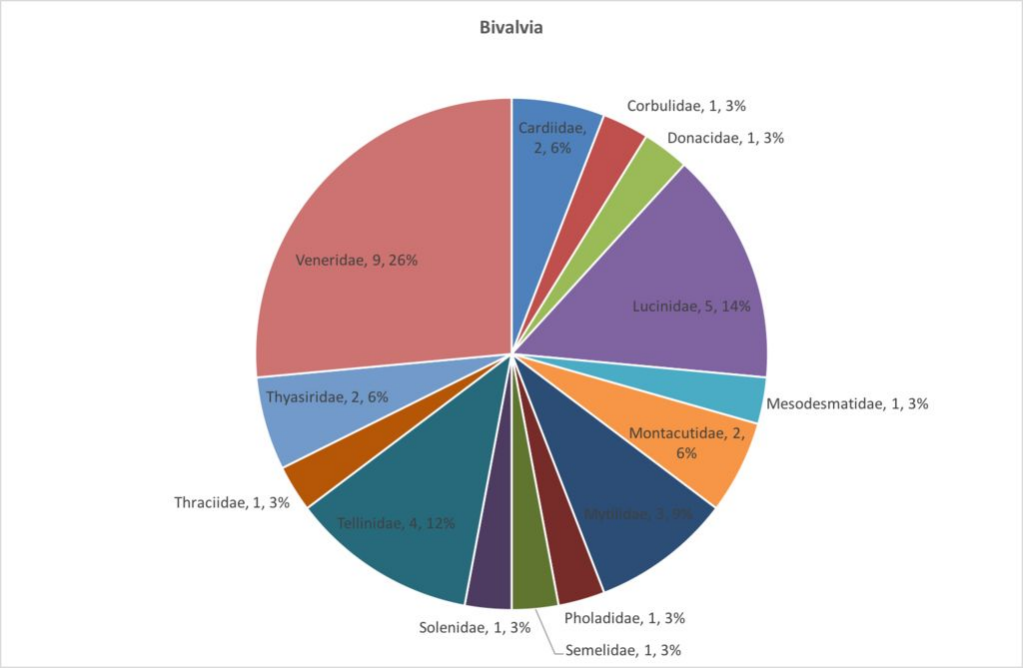
Distribution of species number in the bivalve families.

**Figure 3.**
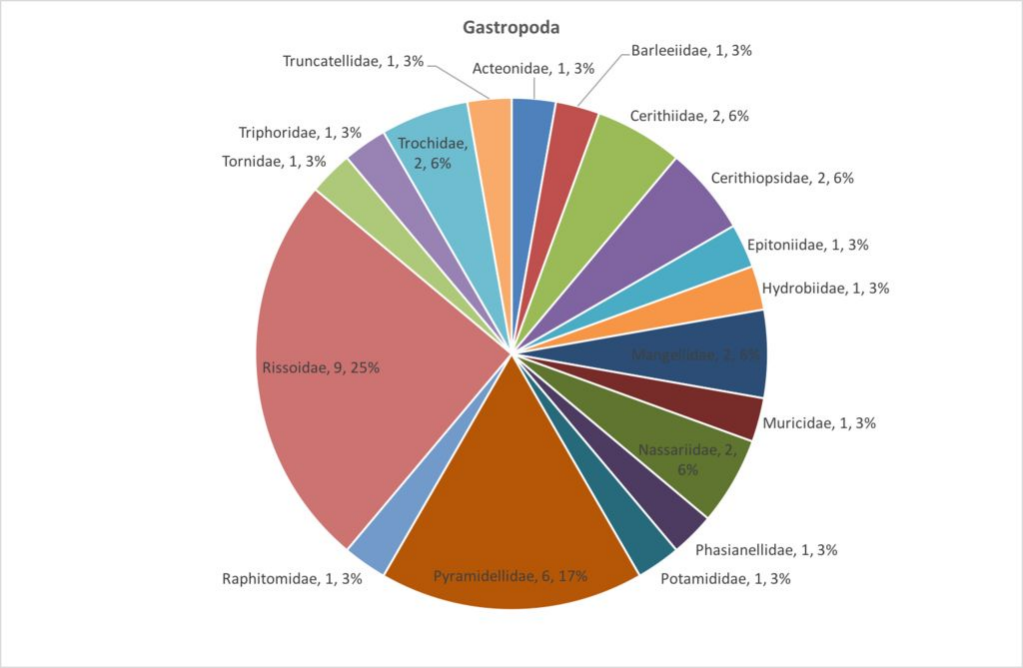
Distribution of species number in the gastropod families.

The distribution of species number in the bivalve and gastropod families is presented in Fig. 2 and Fig. 3 respectively. *Fustiaria rubescens* (Deshayes, 1825), which belongs to the Fustiariidae family, is the only species of tusk shells included in the dataset.

### Taxa included

**Table:** 

Rank	Scientific Name	Common Name
phylum	Mollusca	Molluscs﻿
class	Gastropoda	Gastropods
class	Bivalvia	Bivalves
class	Scaphopoda	Tusk shells

## Temporal coverage

**Single date:** 2004 2 24; 2004 5 28; 2004 9 17; 2004 11 27.

### Notes

The dataset has a temporal coverage of one year (2004): The surveys were seasonal and carried out in Winter (24/2), Spring (28/5), Summer (17/9) and Autumn (27/11). 

## Usage rights

### Use license

Open Data Commons Attribution License

## Data resources

### Data package title

Spatial and seasonal variability of the molluscan macrofauna at the marine-lagoonal environmental gradient at Kalloni saltworks (Lesvos Island, NE Aegean Sea, Greece)

### Resource link

http://ipt.medobis.eu/resource?r=kalloni_saltworks_phd

### Number of data sets

1

### Data set 1.

#### Data set name

Spatial and seasonal variability of the molluscan macrofauna at the marine-lagoonal environmental gradient at Kalloni saltworks (Lesvos Island, NE Aegean Sea, Greece)

#### Number of columns

41

#### Description

The dataset includes two files: Events and Occurences. The former contains the information on the sampling design, whereas the latter contains primarily the species abundance, biomass and taxonomy information.

**Data set 1.** 

Column label	Column description
eventID	An identifier for the set of information associated with an Event (something that occurs at a place and time).
samplingProtocol	The name of, reference to, or description of the method or protocol used during an Event.
sampleSizeValue	A numeric value for a measurement of the size (time duration, length, area, or volume) of a sample in a sampling event.
sampleSizeUnit	The unit of measurement of the size (time duration, length, area, or volume) of a sample in a sampling event.
eventDate	The date-time or interval during which an Event occurred.
year	The four-digit year in which the Event occurred, according to the Common Era Calendar.
month	The ordinal month in which the Event occurred.
day	The integer day of the month on which the Event occurred.
habitat	A category or description of the habitat in which the Event occurred.
fieldNumber	An identifier given to the event in the field.
locationID	An identifier for the set of location information.
locality	The specific description of the place.
minimumDepthInMeters	The lesser depth of a range of depth below the local surface, in meters.
maximumDepthInMeters	The greater depth of a range of depth below the local surface, in meters.
locationRemarks	Comments or notes about the Location.
decimalLatitude	The geographic latitude (in decimal degrees, using the spatial reference system given in geodeticDatum) of the geographic center of a Location. Positive values are north of the Equator, negative values are south of it.
decimalLongitude	The geographic longitude (in decimal degrees, using the spatial reference system given in geodeticDatum) of the geographic center of a Location. Positive values are east of the Greenwich Meridian, negative values are west of it.
coordinateUncertaintyInMeters	The horizontal distance (in meters) from the given decimalLatitude and decimalLongitude describing the smallest circle containing the whole of the Location.
institutionCode	The name (or acronym) in use by the institution having custody of the object(s) or information referred to in the record.
collectionCode	The name, acronym, coden, or initialism identifying the collection or data set from which the record was derived.
basisOfRecord	The specific nature of the data record.
occurenceID	An identifier for the Occurrence.
catalogNumber	An identifier for the record within the data set or collection.
individualCount	The number of individuals represented present at the time of the Occurrence.
organismQuantity	A number or enumeration value for the quantity of organisms.
organismQuantityType	The type of quantification system used for the quantity of organisms.
lifeStage	The age class or life stage of the biological individual(s) at the time the Occurrence was recorded.
identifiedBy	A list (concatenated and separated) of names of people, groups, or organizations who assigned the Taxon to the subject.
scientificNameID	An identifier for the nomenclatural (not taxonomic) details of a scientific name.
scientificName	The full scientific name, with authorship and date information if known.
kingdom	The full scientific name of the kingdom in which the taxon is classified.
phylum	The full scientific name of the phylum or division in which the taxon is classified.
class	The full scientific name of the class in which the taxon is classified.
order	The full scientific name of the order in which the taxon is classified.
family	The full scientific name of the family in which the taxon is classified.
genus	The full scientific name of the family in which the taxon is classified.
subgenus	The full scientific name of the subgenus in which the taxon is classified.
specificEpithet	The name of the first or species epithet of the scientificName.
scientificNameAuthorship	The authorship information for the scientificName formatted according to the conventions of the applicable nomenclaturalCode.
nomenclaturalCode	The nomenclatural code (or codes in the case of an ambiregnal name) under which the scientificName is constructed.
taxonRemarks	Comments or notes about the taxon or name.

## Additional information

### Resource citation

Evagelopoulos A (2015): Spatial and seasonal variability of the molluscan macrofauna at the marine-lagoonal environmental gradient at Kalloni saltworks (Lesvos Island, NE Aegean Sea, Greece). v1.7. Hellenic Center for Marine Research. Dataset/Samplingevent. http://ipt.medobis.eu/resource?r=kalloni_saltworks_phd&v=1.7

### Abundance and biomass variability

**Figure 4.**
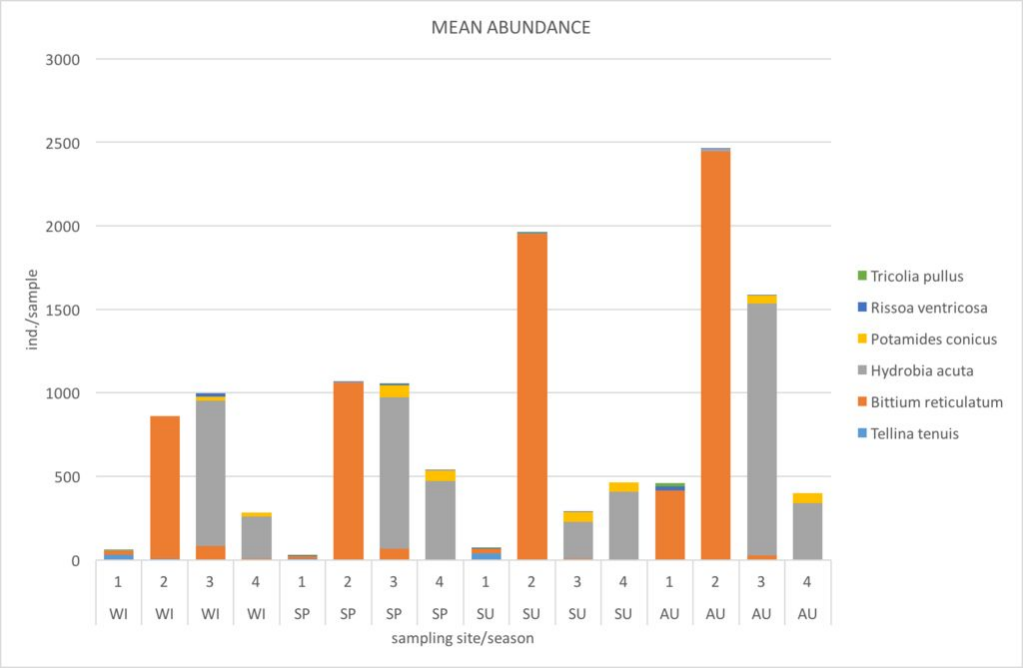
C﻿ontributions of the most important species in the mean total abundance (ind./sample) for each site (1, 2, 3, 4) and seasonal survey (WI: winter, SP: spring, SU: summer, AU: autumn). The most important species in terms of abundance were selected as the ones contributing in at least 10% of the total mean abundance in a dataset sample.

The contributions of the most important species in the mean total abundance (ind./sample) for each site and seasonal survey are given in Fig. 4​.

The most important species in terms of abundanceduring all surveys, were the cerithiid gastropod *Bittium reticulatum* at site 2 and the mud snail *Hydrobia acuta* at sites 3 and 4.

**Figure 5.**
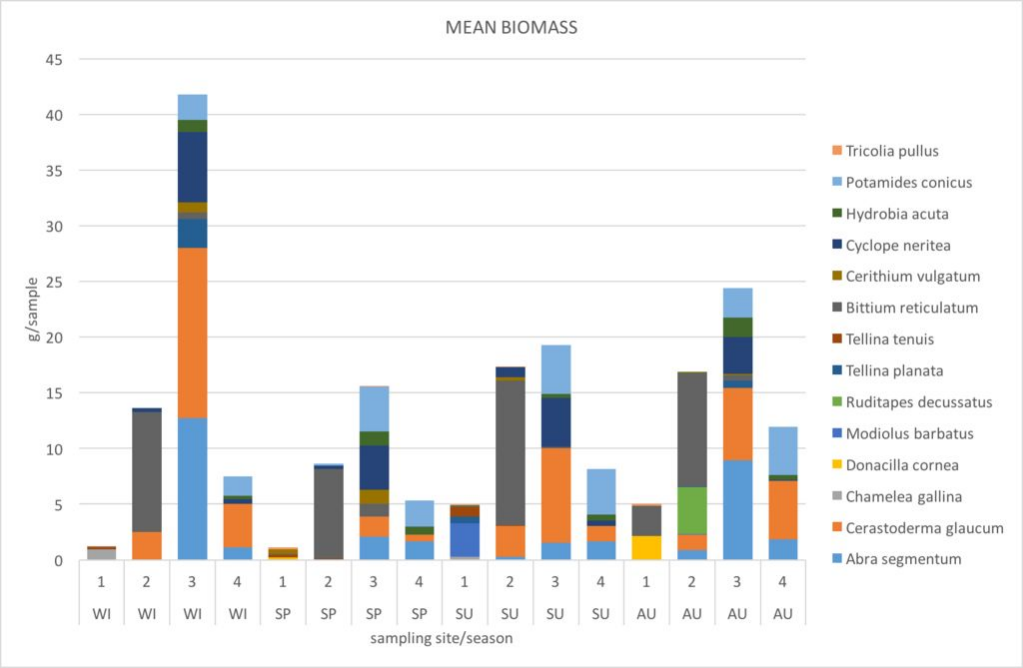
Contributions of the most important species in the mean total biomass (g/sample) for each site (1, 2, 3, 4) and seasonal survey (WI: winter, SP: spring, SU: summer, AU: autumn) . The most important species in terms of biomass were selected as the ones contributing in at least 10% of the total mean biomass in a dataset sample.

The contributions of the most important species in the mean total biomass (g/sample) for each site and seasonal survey are presented in ​Fig. 5.

In terms of biomass, the most important species at site 2 was, during all surveys, the cerithiid gastropod *Bittium reticulatum, *whereas the most important species at sites 3 and 4  were, during all surveys, the bivalves *Abra segmentum* and *Cerastoderma glaucum* and the gastropods *Cyclope neritea* and *Potamides conicus*.

### Observed species diversity variability

**Figure 6.**
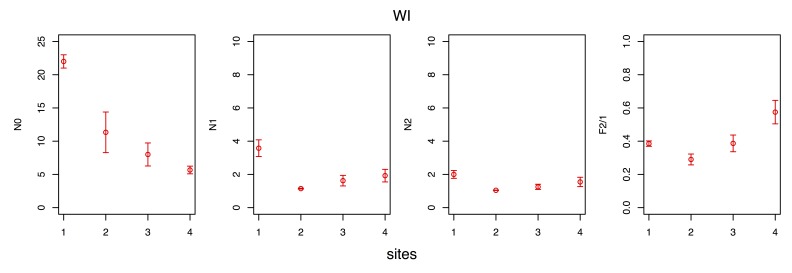
Variability of the observed species diversity and eveness along the sites (1, 2, 3, 4) transect during the winter (WI) survey. Diversity was measured using the Hill numbers N0, N1 and N2, whereas eveness was measured with the F2/1 index.

**Figure 7.**
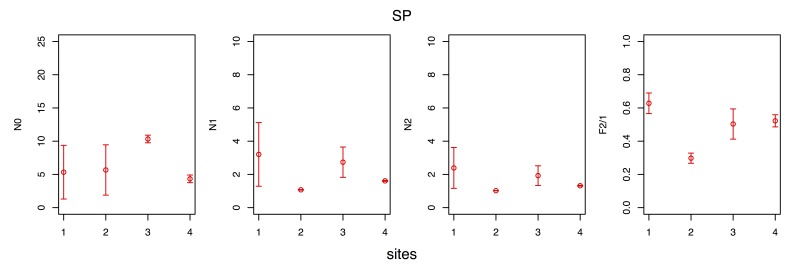
Variability of the observed species diversity and eveness along the sites (1, 2, 3, 4) transect during the spring (SP) survey. Diversity was measured using the Hill numbers N0, N1 and N2, whereas eveness was measured with the F2/1 index.

**Figure 8.**
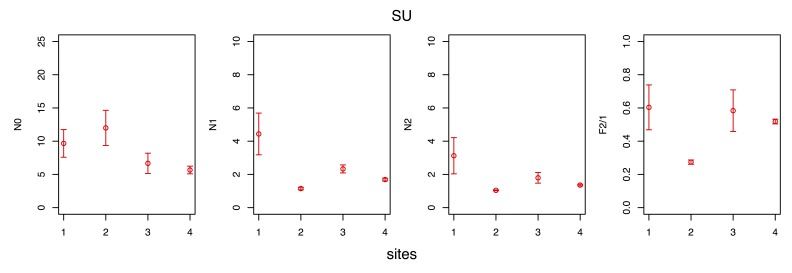
Variability of the observed species diversity and eveness along the sites (1, 2, 3, 4) transect during the summer (SU) survey. Diversity was measured using the Hill numbers N0, N1 and N2, whereas eveness was measured with the F2/1 index.

**Figure 9.**
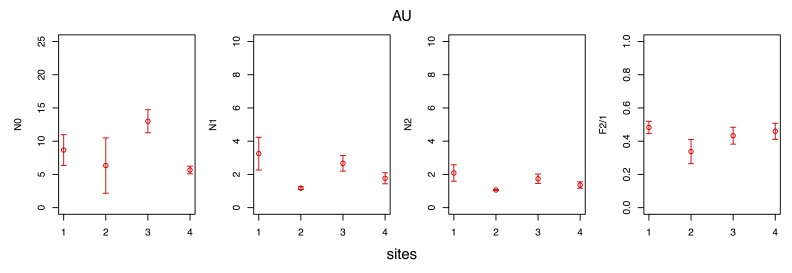
Variability of the observed species diversity and eveness along the sites (1, 2, 3, 4) transect during the autumn (AU) survey. Diversity was measured using the Hill numbers N0, N1 and N2, whereas eveness was measured with the F2/1 index.

The spatial variability of the observed species diversity and eveness during each seasonal survey are given in Figs 6, 7, 8, 9. 

Observed species diversity was measured with the Hill numbers (Hill 1973 ) N0 (= species richness), N1 (= the exponential form of the Shannon-Wiener entropy index) and N2 (= the reciprocal form of the Simpson concentration index), whereas eveness was measured using the F2/1 eveness index (Alatalo 1981), a transformation of the N2/1 eveness index of Hill (Hill 1973). All the aforementioned indices have several desirable properties and are preferable to the classic diversity (e.g. the Shannon-Wiener index) and eveness (e.g. the Pielou index) indices (Jost 2006, Tuomisto 2010, Tuomisto 2012).

The maximum observed species richness (N0 index) was measured either at site 1, 2 or 3, depending on the season, whereas the minimum was invariably measured at site 4. The minimum observed diversity (N1, N2 indices) was invariably recorded at site 2, due to the minimum of eveness measured therein (F2/1 index).

## Supplementary Material

Supplementary material 1 The dataset Data type: abundance, biomass and taxonomic classification
Brief description: The dataset file includes the abundance (ind./sample), wet biomass with shells (g/sample) and taxonomic classification of the species in each sample.
File: oo_108852.xlsx Athanasios Evagelopoulos

## Figures and Tables

**Table 1.** Geographic coordinates of the sampling sites.

Sampling site	Site description	Latitude (DD)	Longitude (DD)
1	Kalloni Gulf	39.204974	26.250093
2	Inlet channel	39.207020	26.251612
3	Pond 1	39.209713	26.253069
4	Pond 2	39.208540	26.258517

**Table 2.** List of species included in the dataset, indicating their taxonomic classification.

**Scientific name**	**Authority**	**Class**	**Family**
*Abra segmentum*	(Récluz, 1843)	Bivalvia	Semelidae
*Axinulus croulinensis*	(Jeffreys, 1847)	Bivalvia	Thyasiridae
*Cerastoderma glaucum*	(Bruguière, 1789)	Bivalvia	Cardiidae
*Chamelea gallina*	(Linnaeus, 1758)	Bivalvia	Veneridae
*Clausinella fasciata*	(da Costa, 1778)	Bivalvia	Veneridae
*Corbula gibba*	(Olivi, 1792)	Bivalvia	Corbulidae
*Ctena decussata*	(O. G. Costa, 1829)	Bivalvia	Lucinidae
*Lucinella divaricata*	(Linnaeus, 1758)	Bivalvia	Lucinidae
*Donacilla cornea*	(Poli, 1791)	Bivalvia	Mesodesmatidae
*Donax venustus*	﻿Poli, 1795	Bivalvia	Donacidae
*Gouldia minima*	(Montagu, 1803)	Bivalvia	Veneridae
*Irus irus*	(Linnaeus, 1758)	Bivalvia	Veneridae
*Loripes lucinalis*	(Lamarck, 1818)	Bivalvia	Lucinidae
*Loripinus fragilis*	(Philippi, 1836)	Bivalvia	Lucinidae
*Gibbomodiola adriatica*	(Lamarck, 1819)	Bivalvia	Mytilidae
*Modiolus barbatus*	(Linnaeus, 1758)	Bivalvia	Mytilidae
*Montacuta substriata*	(Montagu, 1808)	Bivalvia	Montacutidae
*Musculus* sp.	Röding, 1798	Bivalvia	Mytilidae
*Myrtea spinifera*	(Montagu, 1803)	Bivalvia	Lucinidae
*Kurtiella bidentata*	(Montagu, 1803)	Bivalvia	Montacutidae
*Parvicardium exiguum*	(Gmelin, 1791)	Bivalvia	Cardiidae
*Pholas dactylus*	Linnaeus, 1758	Bivalvia	Pholadidae
*Pitar rudis*	(Poli, 1795)	Bivalvia	Veneridae
*Solen marginatus*	Pulteney, 1799	Bivalvia	Solenidae
*Polititapes aureus*	(Gmelin, 1791)	Bivalvia	Veneridae
*Ruditapes decussatus*	(Linnaeus, 1758)	Bivalvia	Veneridae
*Moerella donacina*	(Linnaeus, 1758)	Bivalvia	Tellinidae
*Tellina planata*	Linnaeus, 1758	Bivalvia	Tellinidae
*Tellina* sp.	Linnaeus, 1758	Bivalvia	Tellinidae
*Tellina tenuis*	da Costa, 1778	Bivalvia	Tellinidae
*Thracia phaseolina*	(Lamarck, 1818)	Bivalvia	Thraciidae
*Thyasira flexuosa*	(Montagu, 1803)	Bivalvia	Thyasiridae
*Timoclea ovata*	(Pennant, 1777)	Bivalvia	Veneridae
*Venus casina*	Linnaeus, 1758	Bivalvia	Veneridae
*Acteon tornatilis*	(Linnaeus, 1758)	Gastropoda	Acteonidae
*Barleeia unifasciata*	(Montagu, 1803)	Gastropoda	Barleeiidae
*Bela nebula*	(Montagu, 1803)	Gastropoda	Mangeliidae
*Bittium reticulatum*	(da Costa, 1778)	Gastropoda	Cerithiidae
*Cerithiopsis* sp.	Forbes & Hanley, 1850	Gastropoda	Cerithiopsidae
*Cerithiopsis tubercularis*	(Montagu, 1803)	Gastropoda	Cerithiopsidae
*Cerithium vulgatum*	Bruguière, 1792	Gastropoda	Cerithiidae
*Chrysallida* sp.	Carpenter, 1856	Gastropoda	Pyramidellidae
*Circulus* sp.	Jeffreys, 1865	Gastropoda	Tornidae
*Cyclope neritea*	(Linnaeus, 1758)	Gastropoda	Nassariidae
*Ebala pointeli*	(de Folin, 1868)	Gastropoda	Pyramidellidae
*Epitonium clathrus*	(Linnaeus, 1758)	Gastropoda	Epitoniidae
*Gibbula albida*	(Gmelin, 1791)	Gastropoda	Trochidae
*Gibbula ardens*	(Salis Marschlins, 1793)	Gastropoda	Trochidae
*Hydrobia acuta*	(Draparnaud, 1805)	Gastropoda	Hydrobiidae
*Mangelia attenuata*	(Montagu, 1803)	Gastropoda	Mangeliidae
*Nassarius incrassatus*	(Strøm, 1768)	Gastropoda	Nassariidae
*Megastomia conoidea*	(Brocchi, 1814)	Gastropoda	Pyramidellidae
*Potamides conicus*	(Blainville, 1829)	Gastropoda	Potamididae
*Pusillina marginata*	(Michaud, 1830)	Gastropoda	Rissoidae
*Pusillina radiata*	(Philippi, 1836)	Gastropoda	Rissoidae
*Raphitoma echinata*	(Brocchi, 1814)	Gastropoda	Raphitomidae
*Rissoa guerinii*	Récluz, 1843	Gastropoda	Rissoidae
*Rissoa monodonta*	Philippi, 1836	Gastropoda	Rissoidae
*Rissoa membranacea*	(J. Adams, 1800)	Gastropoda	Rissoidae
*Rissoa lia*	(Monterosato, 1884)	Gastropoda	Rissoidae
*Rissoa splendida*	Eichwald, 1830	Gastropoda	Rissoidae
*Rissoa variabilis*	(Von Mühlfeldt, 1824)	Gastropoda	Rissoidae
*Rissoa ventricosa*	Desmarest, 1814	Gastropoda	Rissoidae
*Tricolia pullus*	(Linnaeus, 1758)	Gastropoda	Phasianellidae
*Monophorus perversus*	(Linnaeus, 1758)	Gastropoda	Triphoridae
*Trophonopsis muricata*	(Montagu, 1803)	Gastropoda	Muricidae
*Truncatella subcylindrica*	(Linnaeus, 1767)	Gastropoda	Truncatellidae
*Turbonilla delicata*	Monterosato, 1874	Gastropoda	Pyramidellidae
*Turbonilla lactea*	(Linnaeus, 1758)	Gastropoda	Pyramidellidae
*Pyrgostylus striatulus*	(Linnaeus, 1758)	Gastropoda	Pyramidellidae
*Fustiaria rubescens*	(Deshayes, 1825)	Scaphopoda	Fustiariidae

## References

[B3465635] Alatalo Rauno V. (1981). Problems in the Measurement of Evenness in Ecology. Oikos.

[B2792731] Barnes R. S.K. (1980). Coastal Lagoons: The Natural History of a Neglected Habitat.

[B2872809] Barnes R. S.K., Kjerfve B. (1994). Macrofaunal Community Structure and Life Histories in Coastal Lagoons. Coastal Lagoon Processes.

[B2783235] Basset Alberto, Barbone Enrico, Elliott Michael, Li Bai-Lian, Jorgensen Sven Eric, Lucena-Moya Paloma, Pardo Isabel, Mouillot David (2012). A unifying approach to understanding transitional waters: Fundamental properties emerging from ecotone ecosystems. Estuarine, Coastal and Shelf Science.

[B2796137] Britton R. H., Johnson A. R. (1987). An ecological account of a Mediterranean salina: The Salin de Giraud, Camargue (S. France). Biological Conservation.

[B3465687] Buttigieg Pier, Morrison Norman, Smith Barry, Mungall Christopher J, Lewis Suzanna E, Consortium the ENVO (2013). The environment ontology: contextualising biological and biomedical entities. Journal of Biomedical Semantics.

[B3465675] Buttigieg Pier Luigi, Pafilis Evangelos, Lewis Suzanna E, Schildhauer Mark P, Walls Ramona L, Mungall Christopher J (2016). The environment ontology in 2016: bridging domains with increased scope, semantic density, and interoperation.. Journal of biomedical semantics.

[B2902423] de Wit R., Grillo O., Venire G. (2011). Biodiversity of Coastal Lagoon Ecosystems and Their Vulnerability to Global Change. Ecosystems Biodiversity.

[B2775709] Eleftheriou A., Moore D. C., Eleftheriou A., McIntyre A. (2005). Macrofauna Techniques. Methods for the Study of Marine Benthos.

[B2823032] Evagelopoulos Athanasios, Koutsoubas Drosos (2008). Seasonal community structure of the molluscan macrofauna at the marine‐lagoonal environmental transition at Kalloni solar saltworks (Lesvos Island, NE Aegean Sea, Greece). Journal of Natural History.

[B2872707] Evagelopoulos Athanasios, Koutsoubas Drosos, Basset Alberto, Pinna Maurizio, Dimitriadis Charalampos, Sangiorgio Franca, Barbone Enrico, Maidanou Maria, Koulouri Panayota, Dounas Costas (2008). Spatial and seasonal variability of the macrobenthic fauna in Mediterranean solar saltworks ecosystems. Aquatic Conservation: Marine and Freshwater Ecosystems.

[B2773669] Evagelopoulos Α. (2008). Πρότυπα βιοποικιλότητας στα παράκτια υγροτοπικά οικοσυστήματα των αλυκών Καλλονής Λέσβου.

[B2795304] Guelorget O., Perthuisot J. P. (1992). Paralic ecosystems. Biological organization and functioning.. Vie et Milieu.

[B3465625] Hill M. O. (1973). Diversity and Evenness: A Unifying Notation and Its Consequences. Ecology.

[B3465645] Jost Lou (2006). Entropy and diversity. Oikos.

[B2823022] Koutsoubas D., Arvanitidis C., Dounas C., Drummond L. (2000). Community structure and dynamics of the molluscan fauna in a Mediterranean lagoon (Gialova lagoon, SW Greece). Belgian Journal of Zoology.

[B2791974] Levin Lisa A., Boesch Donald F., Covich Alan, Dahm Cliff, Erséus Christer, Ewel Katherine C., Kneib Ronald T., Moldenke Andy, Palmer Margaret A., Snelgrove Paul, Strayer David, Weslawski Jan Marcin (2001). The Function of Marine Critical Transition Zones and the Importance of Sediment Biodiversity. Ecosystems.

[B2792204] Little C. (2000). The biology of shoft shores and estuaries.

[B2823012] Pavlova Penka, Markova Kostadinka, Tanev Stephan, Davis Joseph S. (1998). Observations on a solar saltworks near Burgas, Bulgaria. International Journal of Salt Lake Research.

[B2872745] Pickett S. T. A., Cadenasso M. L. (1995). Landscape Ecology: Spatial Heterogeneity in Ecological Systems. Science.

[B2872725] Reizopoulou Sofia, Nicolaidou Artemis (2004). Benthic diversity of coastal brackish-water lagoons in western Greece. Aquatic Conservation: Marine and Freshwater Ecosystems.

[B2872797] Rossi Francesco, Castelli Alberto, Lardicci Claudio (2006). Distribution of macrobenthic assemblages along a marine gradient in Mediterranean eutrophic coastal lagoons. Marine Ecology.

[B3465665] Tuomisto Hanna (2010). A consistent terminology for quantifying species diversity? Yes, it does exist. Oecologia.

[B3465655] Tuomisto Hanna (2012). An updated consumer’s guide to evenness and related indices. Oikos.

[B2872788] Turner M. G., Gardner R. H. (2015). Landscape Ecology in Theory and Practice: Pattern and Process.

[B2796006] Vieira N., Amat F. (1996). The invertebrate benthic community of two solar salt ponds in Aveiro, Portugal. International Journal of Salt Lake Research.

[B2872735] Wiens John A., Crawford Clifford S., Gosz James R. (1985). Boundary Dynamics: A Conceptual Framework for Studying Landscape Ecosystems. Oikos.

